# Identification of Preterm Labor Evaluation Visits and Extraction of Cervical Length Measures from Electronic Health Records Within a Large Integrated Health Care System: Algorithm Development and Validation

**DOI:** 10.2196/37896

**Published:** 2022-09-06

**Authors:** Fagen Xie, Nehaa Khadka, Michael J Fassett, Vicki Y Chiu, Chantal C Avila, Jiaxiao Shi, Meiyu Yeh, Aniket Kawatkar, Nana A Mensah, David A Sacks, Darios Getahun

**Affiliations:** 1 Department of Research and Evaluation Kaiser Permanente Southern California Pasadena, CA United States; 2 Department of Obstetrics & Gynecology Kaiser Permanente West Los Angeles Medical Center Los Angeles, CA United States; 3 Department of Clinical Science Kaiser Permanente Bernard J Tyson School of Medicine Pasadena, CA United States; 4 Department of Health Systems Science Kaiser Permanente Bernard J. Tyson School of Medicine Pasadena, CA United States

**Keywords:** preterm labor, preterm birth, fetal fibronectin, transvaginal ultrasound, cervical length, natural language processing, computerized algorithm, data extraction, patient records, clinical notes, evaluation notes, patient care, patient notes, electronic health records

## Abstract

**Background:**

Preterm birth (PTB) represents a significant public health problem in the United States and throughout the world. Accurate identification of preterm labor (PTL) evaluation visits is the first step in conducting PTB-related research.

**Objective:**

We aimed to develop a validated computerized algorithm to identify PTL evaluation visits and extract cervical length (CL) measures from electronic health records (EHRs) within a large integrated health care system.

**Methods:**

We used data extracted from the EHRs at Kaiser Permanente Southern California between 2009 and 2020. First, we identified triage and hospital encounters with fetal fibronectin (fFN) tests, transvaginal ultrasound (TVUS) procedures, PTL medications, or PTL diagnosis codes within 24^0/7^-34^6/7^ gestational weeks. Second, clinical notes associated with triage and hospital encounters within 24^0/7^-34^6/7^ gestational weeks were extracted from EHRs. A computerized algorithm and an automated process were developed and refined by multiple iterations of chart review and adjudication to search the following PTL indicators: fFN tests, TVUS procedures, abdominal pain, uterine contractions, PTL medications, and descriptions of PTL evaluations. An additional process was constructed to extract the CLs from the corresponding clinical notes of these identified PTL evaluation visits.

**Results:**

A total of 441,673 live birth pregnancies were identified between 2009 and 2020. Of these, 103,139 pregnancies (23.35%) had documented PTL evaluation visits identified by the computerized algorithm. The trend of pregnancies with PTL evaluation visits slightly decreased from 24.41% (2009) to 17.42% (2020). Of the first 103,139 PTL visits, 19,439 (18.85%) and 44,423 (43.97%) had an fFN test and a TVUS, respectively. The percentage of first PTL visits with an fFN test decreased from 18.06% at 24^0/7^ gestational weeks to 2.32% at 34^6/7^ gestational weeks, and TVUS from 54.67% at 24^0/7^ gestational weeks to 12.05% in 34^6/7^ gestational weeks. The mean (SD) of the CL was 3.66 (0.99) cm with a mean range of 3.61-3.69 cm that remained stable across the study period. Of the pregnancies with PTL evaluation visits, the rate of PTB remained stable over time (20,399, 19.78%). Validation of the computerized algorithms against 100 randomly selected records from these potential PTL visits showed positive predictive values of 97%, 94.44%, 100%, and 96.43% for the PTL evaluation visits, fFN tests, TVUS, and CL, respectively, along with sensitivity values of 100%, 90%, and 90%, and specificity values of 98.8%, 100%, and 98.6% for the fFN test, TVUS, and CL, respectively.

**Conclusions:**

The developed computerized algorithm effectively identified PTL evaluation visits and extracted the corresponding CL measures from the EHRs. Validation against this algorithm achieved a high level of accuracy. This computerized algorithm can be used for conducting PTL- or PTB-related pharmacoepidemiologic studies and patient care reviews.

## Introduction

Preterm birth (PTB, the birth of a child before 37^0/7^ weeks of gestation) occurs in nearly 10% of live births in the United States [1,2]. It is one of the leading causes of infant morbidity and mortality in the United States and throughout the world [3,4] and constitutes a significant public health burden [2]. The majority of PTBs are spontaneous or idiopathic, whereas the remaining are medically indicated due to fetal or maternal complications [5-7]. Surviving infants are at significantly increased risk for long-term sequelae, including respiratory, gastrointestinal, central nervous system, hearing, and vision problems, as well as long-term cognitive, motor, and behavioral delays with long-lasting effects [2].

The identification of pregnant women at high risk for imminent spontaneous PTB (sPTB) is critical for appropriate and timely management of preterm labor (PTL), including timely administration of antenatal corticosteroids and magnesium sulfate for accelerating fetal lung maturation and neuroprotection [8-11]. On the other hand, accurate assessment of the risk of sPTB including cervical examination and observation of clinical signs and symptoms can allow for better timing of antenatal corticosteroid administration, avoid unnecessary interventions, and reduce costs. Fetal fibronectin (fFN) testing [12] and transvaginal ultrasound (TVUS) measurement of the cervical length (CL) prior to 24 weeks [13] have been used as indicators of potential sPTB risk. For instance, a CL measuring over 3 cm [14] or a negative fFN test [15] obtained from a pregnant woman with presumed PTL may rule against PTL and therefore avoid overdiagnosis and unnecessary treatment. Standardized clinical procedures for the assessment and management of pregnant women with suspected signs and symptoms of PTL have been established [16,17], and although not widely implemented, they have shown significant health care cost reduction by avoiding unnecessary hospitalization of pregnant women who may have signs and symptoms of PTL but are not likely to deliver prematurely [18].

One historical challenge in the evaluation of retrospective patient data has been with respect to the ability to incorporate some of these free-text elements in the electronic health record (EHR); despite being rich sources of data, they have been challenging to incorporate into studies without reliable, consistent, and efficient ways to identify these elements and classify them in data analyses. Natural language processing (NLP) is a field of computer-based methods aimed at standardizing and analyzing free text, for allowing inclusion of these free-text data elements even in large data sets [19-23]. It converts medical information residing in natural language into a more structured format for various medical research and patient care management purposes [24-27]. Although there have been fruitful attempts to predict the risk of sPTB [12-15,28,29] with structured EHRs or machine learning approaches, to our knowledge, there is no available automated algorithm to identify PTL evaluation visits among patients presenting at triage or hospitals from the EHR. The ability to examine all cases of threatened PTL in a large data set, their associated methods of evaluation, and their outcomes and costs will ultimately help inform the discussion surrounding the standardization of threatened PTL assessment and the associated use of TVUS and fFN. The purpose of the present study was to develop and validate a computerized NLP algorithm and process to effectively identify PTL evaluation visits and extract corresponding CL data from the EHRs, including free-text clinical notes, within a large integrated health care system.

## Methods

### Study Setting and Population

Kaiser Permanente Southern California (KPSC) is a large integrated health care system providing comprehensive medical services to over 4.7 million members across 15 large medical center areas. The demographic characteristics of KPSC members are diverse and largely representative of the residents in Southern California [30] with health insurance through group plans, individual plans, Medicare, and Medicaid programs, representing >260 ethnicities and >150 spoken languages. KPSC’s extensive EHR data contain individual-level structured data (including diagnosis codes, procedure codes, medications, immunization records, laboratory results, and pregnancy episodes and outcomes) and unstructured data (including free-text clinical notes, radiology reports, pathology reports, imaging, and videos) covering all medical visits across all health care settings (ie, outpatient, inpatient, emergency department, virtual, etc). Clinical care of KPSC members provided by external contracted providers is captured in the EHR through reimbursement claim requests.

### Ethics Approval

The study protocol was reviewed and approved by the KPSC Institutional Review Board with a waiver of the requirement for informed consent (approval number: 12670). Only authorized persons were given access permission to perform all analyses.

### Identification of PTL Evaluation Visits

The details of PTL assessments are documented in the EHR system in both structured (eg, fFN results, TVUS, and medication) and unstructured (eg, contraction frequency and CL) formats. We conducted a retrospective cohort study including all pregnancies and live births delivered at KPSC hospitals (N=441,673) between 2009 and 2020. The encounters between 24^0/7^ and 34^6/7^ weeks of gestation for each pregnancy episode and the corresponding medical information including clinical notes were extracted from the KPSC EHR system. The extracted information was then used to develop the computerized algorithm and process for identifying PTL evaluation visits through a refined iterative chart review process by the following steps. The encounters between 20^0/7^ and 23^6/7^ weeks as well as those between 35^0/7^ and 36^6/7^ weeks of gestation were excluded because fFN testing was not indicated in these gestational age groups.

Step 1: Based on the codes described in Table A1 of Multimedia Appendix 1, any of the following potential PTL-related encounters for each pregnancy episode were identified and assembled: encounter involving fFN testing, encounter involving TVUS, encounter with PTL diagnosis codes, and encounter with PTL medication.

If any of the above encounters was detected, it was passed to Step 3 for further processing.

Step 2: The evidence or indicator of PTL evaluation was identified from the clinical notes through the following process:

1. Clinical notes associated with triage or hospitalization encounters between 24^0/7^and 34^6/7^ weeks of gestation for each pregnancy episode were extracted, but these were limited to the notes of interest to the study, as shown in Table A2 of Multimedia Appendix 1. Experienced obstetric gynecologists determined these note types.
2. Extracted clinical notes were preprocessed through letter lowercase conversion and sentence separation and tokenization (ie, segmenting text into linguistic units such as words and punctuation) [20]. The separated sentences were further cleaned up by removing the nondigital or nonletter characters except for spaces, periods, commas, and colons, while correcting misspelled words and standardizing abbreviated words or terms detected from the process of algorithm development. The complete corrected and standardized word lists are summarized in Table A3 of Multimedia Appendix 1.
3. Sentences extracted with at least one of the following predefined keywords are listed in Table A4 of Multimedia Appendix 1: preterm labor, fetal fibronectin, transvaginal ultrasound, abdominal pain, and uterine contraction. These keywords of interest to the study were compiled through consultations with experienced obstetric gynecologists. Sentences without any predefined keywords were not passed for further processing.

The following indicators of PTL evaluation were extracted from the above extracted sentences: performed fetal fibronectin test, performed transvaginal ultrasound, abdominal pain, uterine contraction, and explicit descriptions regarding preterm labor evaluation, such as “in preterm labor,” “ruled out PTL,” and “assessment: preterm labor.” Any negated, general, history-related, and uncertain descriptions were excluded.

If any of the above indicators was detected, the corresponding encounter was defined as a PTL evaluation encounter.

Step 3: The PTL evaluation encounters identified in Step 1 and Step 2 were combined, and deduplication was performed if the same encounter was found multiple times. However, encounters with the following conditions were excluded:

1. The encounter was a delivery encounter in patients with the pre-eclampsia/eclampsia diagnosis code. These were excluded due to potential confounding results related to a medically indicated PTB.
2. The encounter had a PTL diagnosis code but without any other evidence of evaluation for PTL in the same encounter (eg, TVUS, uterine contraction, and fFN test). The percentage of this group was relatively small (1.9%). We decided to exclude these potential cases due to the low confirmed rate from the chart review of a randomly selected sample (see the chart review process below).

Step 4: If the identified PTL encounters had an overlapping time window, these encounters were consolidated as a combined PTL encounter, in which the admitted time was the earlier admitted time, whereas the discharge time was the later discharge time.

**Figure 1:**
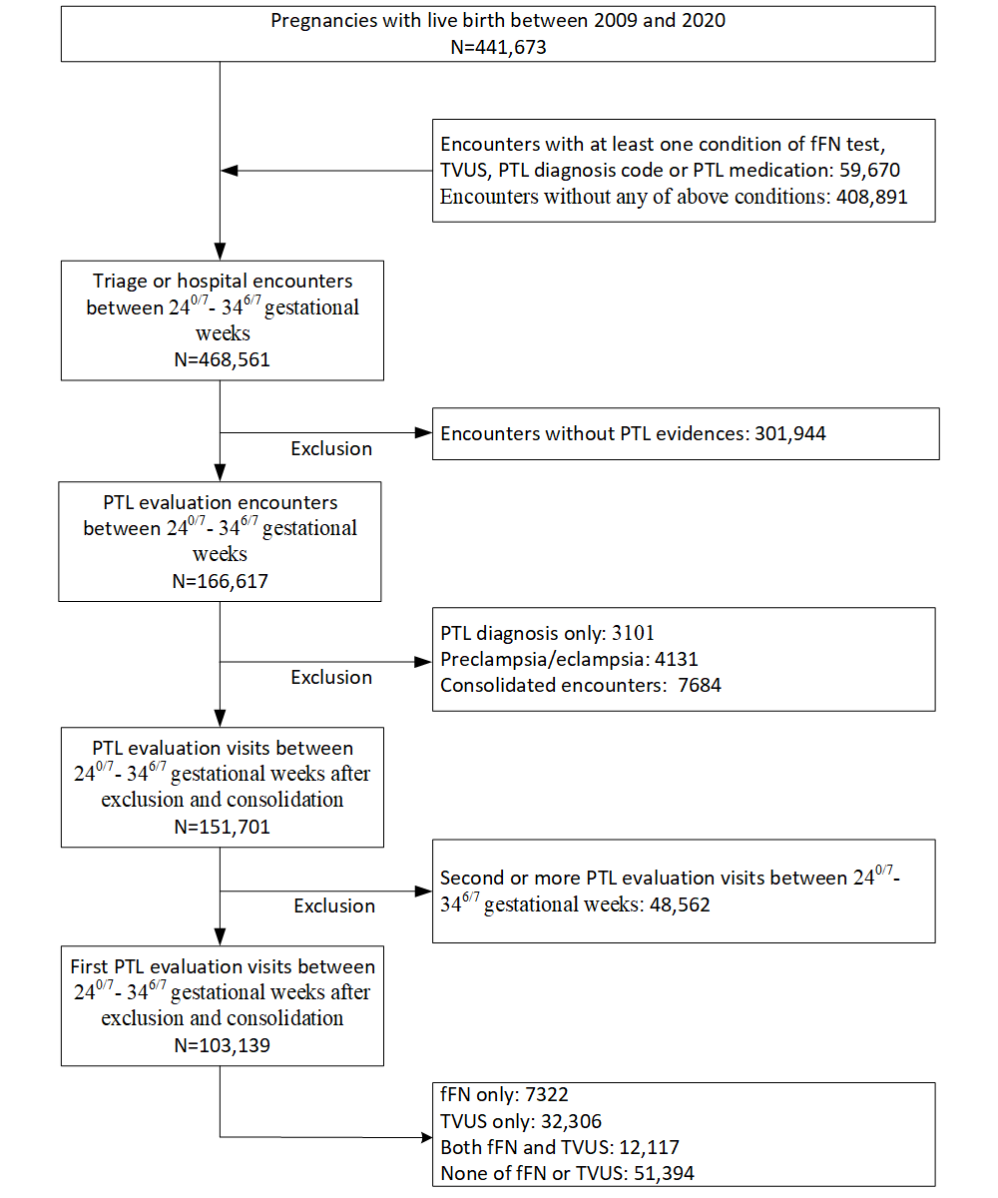
Flowchart showing preterm labor evaluation visits. fFN: fetal fibronectin; PTL: preterm labor; TVUS: transvaginal ultrasound.

Figure 1 presents the number of encounters derived from the process between 24^0/7^ and 34^6/7^ weeks.

### CL Measurement Extraction

Cervical assessment may be performed via transvaginal or transabdominal ultrasound to determine CL during PTL evaluation visits; it can be used as a guide for either admission to the hospital or discharge home, as well as for making management decisions when interpreted in the context of clinical assessment and fFN where possible [16,17]. The measured CL was usually documented in the clinical notes or radiology reports by the examining health care provider. However, retrieving and formatting this measure presented a challenge due to the wide variety of free-text formats used. Therefore, a computerized process was developed to extract CL measures from clinical notes associated with a particular PTL evaluation encounter as in the following steps.

Step 1: Lists of keywords or phrases used to describe CL were compiled based on the knowledge of conventional usage by experienced obstetric gynecologists and enriched by iterative refinement. The complete lists are summarized in Table A5 of Multimedia Appendix 1 and separated into 3 priority groups.

Step 2: The sentences in each clinical note were searched for the preidentified keywords or phrases. If one of the predefined keywords was identified in a sentence, then Step 3 was performed. If no keyword was detected, the search was stopped, and the algorithm moved to the next note.

Step 3: The numeric values associated with the keyword “forward” within 10 tokens in the same sentence were searched starting from the position where the predefined keyword was found. If no values were found during forward searching, then the potentially associated values were searched “backward” within 5 tokens before the keyword position because some values were described before the keyword. However, the extracted value was ignored or excluded if it described other measures rather than the CL, such as cervical dilation. The retrieved measures could be 1 or multiple values or a range of values. In addition, each value could contain a unit (cm or mm) or not have any unit. Examples include “cervical length measures 1.6 cm,” “tvus cl 2.6-2.7 cm no funneling,” “cervical length 3.3 to 4.4,” and “transvaginal ultrasound at bedside 41 mm long cervical length.”

Step 4: The final CL measure was determined for each clinical note based on the keyword or phrase priority. If multiple keywords with different priorities were found in the note, the measured values associated with the keywords with the highest priority were retained. If the retained highest priority group still contained multiple different values, the shortest one was retained.

Step 5: The CL measures were determined for each PTL evaluation visit. A PTL evaluation visit could contain multiple CL values measured at different times. If the encounter was a delivery encounter, the first measure was used as the final CL. Otherwise, the measure closest to discharge was used as the final CL.

Step 6: The CL was standardized and finalized for each PTL evaluation visit. If the measure did not have an associated unit, it was considered cm by default. When the unit was mm/millimeter, the values were divided by 10. Finally, if ranges or multiple values were extracted, then the average value of the extracted values was considered the CL.

### Chart Reviews and Validation Process

To validate the computerized algorithm for identifying true PTL evaluation visits in the EHRs, an iterative chart review process was completed by trained research chart abstractors and adjudicated by experienced obstetrician-gynecologists via multiple iterations. The trained research chart abstractors were provided a spreadsheet with the patients’ unique medical record numbers and visit encounters with the encounter start and end dates. An encounter was considered a true PTL evaluation visit if any of the following criteria were met based on the review of free text in the medical notes: fFN test performed, TVUS performed, clinician description or mention of PTL in the encounter note, clinician description of contractions or abdominal pain in the encounter note, CL obtained, and administration of a PTL-related medication (eg, tocolysis, magnesium sulfate, and corticosteroids).

If any of the evaluation criteria were marked as “yes,” then the encounter was categorized as a PTL evaluation visit. Otherwise, it was not categorized as a PTL evaluation visit. The corresponding supporting information for the decision was documented in detail as well.

First, a sample of 20 encounters was randomly selected from the group with PTL diagnosis codes only but without any other evidence of evaluation for PTL, and the trained research chart abstractors reviewed the chart. Of the 20 encounters, 7 (35%) PTL diagnosis codes were confirmed as PTL evaluation encounters. Due to the low confirmed rate, the encounters with PTL diagnosis only were excluded from further processing. Second, another sample of 20 potential PTL evaluation visits identified by the computerized process was randomly selected for chart review. Among these, 17 (85%) were confirmed as true PTL evaluation visits, and the chart review results were then used for refining and finalizing the process. Finally, 100 potential PTL evaluation visits were randomly selected for full chart review, and the chart review results were used as the reference standard to assess the algorithm's performance to accurately identify true cases of threatened PTL evaluation.

### Data Analysis

Results of PTL evaluation visits, fFN tests, TVUS procedures, and CL measurements generated from the computerized algorithm and process were first evaluated against the chart-reviewed and adjudicated reference standard, including their sensitivity, specificity, and positive predictive value (PPV). Descriptive analyses were then conducted to report the distribution of the first identified PTL evaluation visit of each pregnancy episode by birth year, PTB status, and gestational age in detail. Gestational age at birth was based on the clinical estimate and captured as a structured format in the EHRs.

## Results

A total of 441,673 live birth pregnancy episodes were extracted from the KPSC EHR system from January 1, 2009, to December 31, 2020. Of them, 103,139 (23.35%) were identified by the computerized algorithm and process with at least 1 PTL evaluation visit between 24^0/7^ and 34^6/7^ gestation weeks. The percentage of pregnancies with PTL evaluation visits was stable at approximately 24% between 2009 and 2015 and decreased starting in 2016 (Table 1). The annual trend of PTB associated with PTL among these pregnancies with PTL evaluation visits is shown in Table 2. The overall rate of PTB among pregnant women triaged for PTL evaluation was 19.78% and stable at a range of 18%-20% across the study period.

Table 3 presents the distribution of the identified first PTL evaluation visit of each pregnancy with fFN tests, TVUS procedures, and CL measures by birth year. The rate of the performed fFN tests decreased from 28.33% in 2009 to 9.01% in 2020, whereas the percentage of TVUS procedures increased from 36.72% in 2009 to 45.22% in 2020 and the rate of CL reporting increased from 35.32% in 2009 to 42.36% in 2020. In addition, the rate of PTL with both the fFN test and TVUS procedure decreased from 14.64% in 2009 to 6.85% in 2020. The mean CL was 3.66 cm (SD=0.99 cm) and remained relatively stable over the study period.

Table 4 summarizes the distribution of the identified first PTL evaluation visit of each pregnancy with PTB, fFN tests, TVUS procedures, and CL measurements by the corresponding gestation age at the PTL evaluation visit. For the percentage of patients who ultimately had an sPTB varying by gestational age at the time of assessment, the sPTB decreased from 20.75% in patients presenting at 24^0/7^-24^6/7^ gestational weeks to 16.7% at 27^0/7^-27^6/7^ gestational weeks; it stayed in the range of 16%-19% between 27^0/7^ and 30^6/7^ gestational weeks and then increased from 19.38% at 31^0/7^-31^6/7^ gestational weeks to 24.52% at 34^0/7^-34^6/7^ gestational weeks.

**Table 1:** Trend showing pregnancies resulting in live births with preterm labor evaluation visits within 24^0/7^-34^6/7^ gestational weeks by birth year.

Birth year	Live birth pregnancy, N	Live birth pregnancy with preterm labor evaluation visit, n (%)
2009	31,476	7682 (24.41)
2010	31,388	7798 (24.84)
2011	32,896	8084 (24.57)
2012	34,765	8514 (24.49)
2013	34,968	8477 (24.24)
2014	36,148	8993 (24.88)
2015	37,782	9109 (24.11)
2016	39,605	9486 (23.95)
2017	40,030	9412 (23.51)
2018	41,026	9511 (23.18)
2019	41,326	9061 (21.93)
2020	40,263	7012 (17.42)
Overall	441,673	103,139 (23.35)

**Table 2:** Live birth pregnancies with preterm labor evaluation visits between 24^0/7^ and 34^6/7^ weeks of gestation by birth year and preterm birth status.

Birth year	Preterm birth status		
	Yes^a^, n (%)	No, n (%)	Total (N)
2009	1556 (20.26)	6126 (79.74)	7682
2010	1602 (20.54)	6196 (79.46)	7798
2011	1638 (20.26)	6446 (79.74)	8084
2012	1698 (19.94)	6816 (80.06)	8514
2013	1644 (19.39)	6833 (80.61)	8477
2014	1645 (18.29)	7348 (81.71)	8993
2015	1755 (19.27)	7354 (80.73)	9109
2016	1859 (19.6)	7627 (80.4)	9486
2017	1870 (19.87)	7542 (80.13)	9412
2018	1814 (19.07)	7697 (80.93)	9511
2019	1809 (19.96)	7252 (80.04)	9061
2020	1509 (21.52)	5503 (78.48)	7012
Overall	20,399 (19.78)	82,740 (80.22)	103,139

^a^Yes: preterm births among those pregnancies with preterm labor evaluations.

**Table 3:** First preterm labor evaluation visit of each pregnancy identified by the computerized algorithm between 24^0/7^ and 34^6/7^ weeks of gestation by birth year.

Birth year	Total PTL^a^, N	Yes for fFN^b^, n (%)	Yes^c^ for TVUS^d^, n (%)	Yes for both fFN and TVUS, n (%)	Cervical length	
					n (%)	Mean (SD), cm
2009	7682	2176 (28.33)	2821 (36.72)	1125 (14.64)	2713 (35.32)	3.62 (1.01)
2010	7798	2145 (27.51)	2958 (37.93)	1129 (14.47)	2847 (36.51)	3.63 (1.01)
2011	8084	2223 (27.5)	3221 (39.84)	1233 (15.25)	3131 (38.73)	3.63 (0.99)
2012	8514	2155 (25.31)	3579 (42.04)	1276 (15)	3482 (40.9)	3.64 (0.99)
2013	8477	2106 (24.84)	3846 (45.37)	1349 (15.91)	3685 (43.47)	3.61 (0.99)
2014	8993	1848 (20.55)	4134 (45.97)	1264 (14.05)	3949 (43.91)	3.64 (1.00)
2015	9109	1653 (18.15)	4278 (46.96)	1113 (12.22)	4103 (45.04)	3.69 (1.00)
2016	9486	1470 (15.5)	4269 (45)	991 (10.44)	4097 (43.19)	3.68 (0.99)
2017	9412	1172 (12.45)	4045 (42.98)	803 (8.53)	3881 (40.23)	3.69 (0.96)
2018	9511	1009 (10.61)	4025 (42.32)	714 (7.51)	3805 (40.01)	3.68 (0.98)
2019	9061	850 (9.38)	3976 (43.88)	640 (7.06)	3762 (41.52)	3.70 (0.98)
2020	7012	632 (9.01)	3171 (45.33)	480 (6.85)	2970 (43.36)	3.65 (1.00)
Overall	103,139	19,439 (18.85)	44,423 (43.97)	12,117 (11.75)	42,425 (41.13)	3.66 (0.99)

^a^PTL: preterm labor.

^b^fFN: fetal fibronectin.

^c^Yes: It means that the column contains patient records with documented transvaginal ultrasound assessment or cervical length values.

^d^TVUS: transvaginal ultrasound.

**Table 4:** First preterm labor evaluation visit of each pregnancy identified by the computerized algorithm between 24^0/7^ and 34^6/7^ weeks of gestation by gestational age.

Gestation age of PTL^a^ (weeks)	Total PTL cases, N	PTB^b^ -Yes^c^, n (%)	fFN^d^-Yes, n (%)	TVUS^e^-Yes^f^, n (%)	Both fFN and TVUS -Yes, n (%)	Cervical length	
						n (%)	Mean (SD)
24^0/7^-24^6/7^	7691	1596 (20.75)	1397 (18.16)	4205 (54.67)	1013 (13.17)	4009 (52.13)	3.70 (1.06)
25^0/7^-25^6/7^	7496	2468 (19.58)	1403 (18.72)	3983 (53.14)	971 (12.95)	3813 (50.87)	3.73 (1.03)
26^0/7^-26^6/7^	7923	1392 (17.57)	1524 (19.24)	4060 (51.24)	1037 (13.09)	3894 (49.15)	3.76 (1.01)
27^0/7^-27^6/7^	8122	1356 (16.7)	1733 (21.34)	4186 (51.54)	1143 (14.07)	3995 (49.19)	3.75 (0.97)
28^0/7^-28^6/7^	8417	1562 (18.56)	1771 (21.04)	4220 (50.14)	1166 (13.85)	4060 (48.24)	3.71 (0.98)
29^0/7^-29^6/7^	8823	1535 (17.4)	2032 (23.03)	4229 (50.2)	1290 (14.62)	4262 (48.31)	3,68 (0.97)
30^0/7^-30^6/7^	9224	1709 (18.53)	2114 (22.92)	4436 (48.09)	1279 (13.87)	4274 (46.34)	3.67 (0.94)
31^0/7^-31^6/7^	9932	1925 (19.38)	2446 (24.63)	4638 (46.7)	1475 (14.85)	4492 (45.23)	3.59 (0.97)
32^0/7^-32^6/7^	11,158	2234 (20.02)	2639 (23.65)	4752 (42.59)	1520 (13.62)	4567 (40.93)	3.58 (0.95)
33^0/7^-33^6/7^	11,770	2537 (21.55)	2088 (17.74)	3898 (33.12)	1113 (9.46)	3722 (31.62)	3.50 (0.97)
34^0/7^-34^6/7^	12,583	3085 (24.52)	292 (2.32)	1516 (12.05)	100 (0.8)	1337 (10.63)	3.42 (1.08)
Overall	103,139	20,399 (19.78)	19,439 (18.85)	44,423 (43.97)	12117 (11.75)	42,425 (41.13)	3.66 (0.99)

^a^PTL: preterm labor.

^b^PTB: preterm birth.

^c^Yes: It implies preterm births among those pregnancies with preterm labor evaluations.

^d^fFN: fetal fibronectin.

^e^TVUS: transvaginal ultrasound.

^f^ Yes: It means that the column contains patient records with documented transvaginal ultrasound assessment or cervical length values.

The percentage of PTL evaluation visits with fFN tests, TVUS procedures, and CL measurements also varied over the gestational age at presentation. fFN testing increased from 18.16% at 24^0/7^-24^6/7^ gestational weeks to 24.63% at 31^0/7^-31^6/7^ gestational weeks and then dropped significantly to 2.32% at 34^0/7^-34^6/7^ gestational weeks. In contrast, the percentage decreased from 54.67% at 24^0/7^-24^6/7^ gestational weeks to 12.05% at 34^0/7^-34^6/7^ gestational weeks for TVUS procedures, and 52.13% at 24^0/7^-24^6/7^ gestational weeks to 10.63% at 34^0/7^-34^6/7^ gestational weeks for CL measurements. The mean CL also slightly decreased from 3.7 cm (SD=1.06 cm) at 24^0/7^-24^6/7^ gestational weeks to 3.43 cm (SD=1.08 cm) at 34^0/7^-34^6/7^ gestational weeks. The trend of PTL evaluation with both fFN tests and TVUS procedures by gestational age had a pattern similar to PTL visits with fFN tests.

The validation of 100 randomly selected PTL evaluation visits identified by the computerized algorithm against the manual chart review (which served as the gold standard) is presented in Table 5. Of the 100 PTL evaluation visits identified by the NLP algorithm, 18 PTL evaluations involved fFN tests, 27 involved TVUS procedures, and 28 involved CL measures. Further, 97 of the 100 were confirmed PTL evaluation visits, 17 of 18 had confirmed fFN tests, all 27 had confirmed TVUS procedures, and 27 of 28 had confirmed CL measurements recorded. The computerized algorithm missed 3 PTL evaluation visits with TVUS performed and 3 CL measurements. The algorithm yielded PPVs of 97%, 94.44%, 100%, and 96.43% for PTL evaluation visits, fFN tests, TVUS procedures, and CL measurements, respectively, and sensitivity values of 100%, 90%, and 90%, along with specificity values of 98.8%, 100%, and 98.6% for fFN tests, TVUS procedures, and CL measurements, respectively, as observed in Table 6.

**Table 5:** Validation results of the preterm labor evaluation and cervical length measures extraction algorithm.

Computerized results		Total (N)		Status after chart review		
				Yes, n		No, n
Preterm labor evaluation visits		100		97		3
**Fetal fibronectin test**						
	Yes	18	17		1	
	No	82	0		82	
**Transvaginal ultrasound**						
	Yes	27	27		0	
	No	73	3		70	
**Cervical length**						
	Yes-same value	27	27		0	
	Yes-different value	1	1		0	
	No	72	3		69	

**Table 6:** Performance metrics of the algorithm.

Performance	PPV^a^ (%)	Sensitivity (%)	Specificity (%)
Preterm labor evaluation visit	97	NE^b^	NE
Fetal fibronectin test	94.44	100	98.8
Transvaginal ultrasound	100	90	100
Cervical length	96.43	90	98.6

^a^PPV: positive predictive value.

^b^NE: not estimated.

## Discussion

When pregnant women presented in triage with signs and symptoms of PTL, a PTL assessment was performed, and the details of the assessment were documented and stored in the EHR system in both structured and unstructured formats. In this study, we developed a computerized algorithm and process to identify PTL evaluation visits and extract associated methods of evaluation for threatened PTL, including fFN, TVUS, and CL. This algorithm identified the population of patients who presented with threatened PTL and underwent these associated assessments with high sensitivity and specificity. With this algorithm, 23.35% of pregnancies in the study were identified with PTL evaluation visits within 24^0/7^-34^6/7^ gestational weeks and 19.78% of these pregnancies ultimately led to sPTB. This result is consistent with findings reported in previous studies [18,31,32].

It is worth exploring the details of misclassifications against the manual chart review, although the disagreement between manual chart review and NLP outputs was small. Of the 3 false positive PTL evaluation visits, 1 presented for a scheduled cesarean section at 36 gestational weeks; the visit mentioned uterine contractions, which was one of the conditions used to define PTL. The second case with uterine contractions presented for elective induction of labor at 39 gestational weeks and was not excluded due to the inaccurate estimation of the pregnancy start date. The third case was not excluded because the algorithm detected documented discussion of untreated infection potentially increasing the risk of PTL rather than the true PTL assessment. The algorithm produced only 1 false positive finding for fFN because it wrongly identified the phrase “fFN uninterpretable given a recent sex activity” as a positive fFN result. The algorithm missed 3 TVUS procedures, among which 2 were missed because the terms “vaginal ultrasound” and “formal ultrasound” were used to describe ultrasound for CL measurements, and these were not present in the compiled term list. The other missed case was due to the location of the imaging; TVUS was performed during the regular obstetrician office visit rather than in the hospital triage unit. Additionally, the algorithm incorrectly extracted 1 CL measure but missed 3. The CL measures of the missed cases were falsely excluded because the measures were inaccurately associated with other terms by the algorithm, such as “cervix opening/dilation” or “deepest vertical amniotic fluid pocket.” The incorrect one resulted from the false selection of a measurement performed during the obstetrician office visit rather than as part of the hospital triage service because both were mentioned in the same triage clinical notes.

Clinicians routinely conduct PTL assessments when pregnant women present with signs and symptoms of PTL. Such assessments may help distinguish true PTL cases from false ones, for which the subsequent application of appropriate interventions may improve neonatal outcomes [33]. Conversely, discharges to home for false PTL cases prevent unnecessary hospitalization, as well as unnecessary, costly, and potentially harmful interventions [34]. The current use of CL measures and fFN tests during pregnancy is limited to situations where a negative result can avoid unnecessary interventions. Our study algorithm tried to identify all PTL evaluation visits as long as the performed assessment was detected from clinical notes regardless of whether the encounter resulted in an sPTB or the continuation of the pregnancy. The identified PTL evaluation visits will provide a unique opportunity to explore the association of PTL assessment with fetal outcomes. This approach will also provide us the opportunity to accurately ascertain sPTB outcomes and its impact on successive pregnancies as well as differentiate PTBs by subtypes (sPTB from indicated PTB) in future studies.

In recent years, NLP applications have either embraced machine learning techniques alone or in combination with rule-based NLP [27,35]. Machine learning techniques proved advantageous because they improved accuracy when used in situations where the performance obtained with the existing rule-based algorithms was not satisfactory [36]. This technique has been applied in the prediction of PTBs using structured EHR data [27]. To our knowledge, this is the first NLP approach in the medical field to be used for identifying PTL evaluation visits based on either structured or unstructured data. Future work warrants further research in this area via machine learning approaches to improve the performance in terms of identifying PTL evaluation visits.

Our study has several potential limitations. First, our algorithm relied on the available (structured and unstructured) information and the accuracy of the variable in our EHR system. Although clinical notes are not available for individuals receiving outside care, it is less likely that pregnant women would receive their care elsewhere as long as a pregnancy episode was established in our care system. Second, although PTL visits with concomitant medical indications for preterm delivery were not the focus of the study (no PTL assessment performed, directly admitted for delivery), our algorithm only excluded the PTL visits with pre-eclampsia/eclampsia. Other medical conditions, such as scheduled cesarean sections and medically indicated induction of labor, were not integrated into the exclusion criteria of the algorithm due to relatively small sample sizes. Third, when applying to other health care systems and settings, this specific computerized algorithm may require some modifications due to variations in the format and presentation of clinical notes in different health care settings. Finally, this computerized algorithm was limited by the precompiled search terms and lexicons of interest in screening for potentially relevant clinical notes. It may be enhanced by more extensive and representative chart review samples in future work.

In conclusion, the developed NLP algorithm effectively identified PTL evaluation visits and extracted corresponding methods of evaluation for PTL, including fFN, TVUS, and CL measurements from the EHRs. Validation of this algorithm indicated a high level of accuracy. This NLP algorithm can be used to conduct PTL- or PTB-related pharmacoepidemiologic studies and patient care reviews.

## Appendix

Multimedia Appendix 1 Supplementary tables containing the diagnosis codes, procedure, medication list, and key phrases and terms for the preterm labor evaluation visit algorithm.

## Abbreviations

CL: cervical length
EHR: electronic health record
fFN: fetal fibronectin
KPSC: Kaiser Permanente Southern California
NLP: natural language processing
PPV: positive predictive value
PTB: preterm birth
PTL: preterm labor
sPTB: spontaneous preterm birth
TVUS: transvaginal ultrasound
