# Supplementary material for: Identification of Preterm Labor Evaluation Visits and Extraction of Cervical Length Measures from Electronic Health Records Within a Large Integrated Health Care System: Algorithm Development and Validation
Source: JMIR Med Inform. 2022 Sep 6;10(9):e37896. doi: 10.2196/37896 (PMC9490529; doi:10.2196/37896)
Supplement: Multimedia Appendix 1 [file medinform_v10i9e37896_app1.docx]

Table A1. Details of preterm labor diagnosis codes, transvaginal ultrasound procedure codes, and preterm labor medications.

| Preterm labor diagnosis code | 644, 644.0, 644.00, 644.01, 644.03, 644.1, 644.10, 644.2, 644.20, 644.21, O47.9, O47.00, O47.02, O47.03, O60.00, O60.02, O60.03, O60.10X*, O60.12X*, O60.13X*, O60.14* |
| --- | --- |
| Transvaginal ultrasound procedure code | 76817 |
| Preterm labor medication | Nifedipine^¶^, indomethacin^¶^, magnesium sulfate^¶^, adalat, bene-cin, indameth, indo-lemmon, indocin, indoflex, indomed, procardia |

* Zero or one digital character.

¶ These medications are routinely used.

Table A2. Clinical note types used for identifying preterm labor evaluation visits.

| **Note types** | **Definitions** |
| --- | --- |
| Addendum Notes | Addendum notes |
| Consults | Consultation notes |
| Discharge Planning Progress Notes | Discharge planning progress notes |
| Discharge Summary | Discharge summary notes |
| ED Notes | Emergency department notes |
| ED Provider Notes | Emergency department provider notes |
| ED Provider Triage Notes | Emergency department triage notes |
| EvaluateDX | Diagnosis evaluation notes |
| H&P | History and physical notes |
| Initial Assessments | Initial assessment notes |
| Interval H&P Notes | Interval history and physical notes |
| L&D Delivery Notes | Labor and delivery notes |
| Multi-Discipline Progress Notes | Multi-discipline progress notes |
| Non-MD Discharge Summary Progress Notes | Non-MD discharge summary progress notes |
| OPO Progress Note | Organ procurement organization progress notes |
| OR Nursing | Operating room Nursing notes |
| Procedures | Procedure notes |
| Progress Notes | Progress notes |
| Sign-Out Notes | Provider sing-out notes |
| UC Nurse Notes | Urgent care nurse notes |
| Weekly Summary Progress Note | Weekly summary progress notes |

Table A3. Corrections of misspelled words and standardization of abbreviated words.

| **Original word** | **Corrected or standardized word** |
| --- | --- |
| ptl, pre term labor, pre-term labor | preterm labor |
| Inlabor | in labor |
| ffn | fetal fibronectin |
| u s, ultra sound | ultrasound |
| stransvaginal, tranvaginal, tv, tvs, t v, t/v, tranvag, transvag, tran vag, tran vaginal, trans vag, trans vaginal | transvaginal |
| tvu, tvs, tvus, tvusg, tvutz, tvusn, tvusl, tv us, tv ultrasound, tv sono, t/vu, t/vus, t/vusg, t/vutz, t/vusn, t/vusl, t/v us, t/v ultrasound, t/v sono, | transvaginal ultrasound |
| bs ultrasound, bs us, beside us, bs us, bsu | beside ultrasound |
| bs sono | beside sono |
| endovaginal us | endovaginal ultrasound |
| creat cl, creatinine cl | creatinine clearance |
| cervis, cervizx, cx | cervix |
| cervical, cervic al, cervica, cervicale, cervical, cervicle, cervical, cerviocal, cervival, cervixal | cervical |
| lengeth, lengh, lengt, engtth, lengtyh, lengh | length |
| c l, cl, ccl, cxl, cx l, cvx l, cvxl | cervix length |
| tvcx, t/vcx, tv cx, transvaginal cx | transvaginal cervix |
| tv cl, tvcl, tvs cl, tvscl, tvus cl, tvuscl, t/v cl, t/vcl, t/vs cl, t/vscl, t/vus cl, t/vuscl | transvaginal ultrasound cervix length |
| u scl, uscl | ultrasound cervix length |
| h o, h/o, hx of | history of |
| e o, e/o | evidence of |
| abd | abdominal |
| llq pain | left lower quadrant pain |
| rlq pain | right lower quadrant pain |
| luq pain | left upper quadrant pain |
| ruq pain | right upper quadrant pain |
| uc, ucs, ctx | uterine contraction |
| ptuc, ptucs | patient uterine contraction |
| nge, neg | negative |
| Instructions, tinstructions, insts | instructions |
| gvivne | given |
| Categoryuterine | category uterine |
| deies, deneis, densies | denies |
| Ofneed | of need |
| s sx, s s | sign and symptom |
| cer cl age | cerclage |
| Sonograph | sonography |
| Rangd | ranged |
| Longcervical | long cervical |
| Measurd | measured |
| shortenedr, shortenend, shortenned | shortened |
| shortnening, shortening, shortenening | shortening |
| Normaland | normal and |
| bedsie | bedside |
| breaking | beaking |
| reassuing | reassuring |
| cl osed | closed |
| ptrcaution, precaution, precaution, preacution, prec, precauton, precuation | precaution |
| areinadequate | are inadequate |
| cm | centimeter |
| ml, mm | millimeter |

Table A4. Keywords or phrases used for identifying preterm labor evaluation visits from clinical notes.

| **Category** | **Keywords or phrases*** |
| --- | --- |
| Preterm labor | preterm labor, actively labor, early labor, labor check, labor recheck |
| Fetal Fibronectin | fetal fibronectin, fibronectin fetal |
| Transvaginal ultrasound | transvaginal ultrasound, transvaginal us, transvaginal sono, endovaginal ultrasound, endovaginal us, endovaginal sono |
| Abdominal pain | Abdominal pain, abdominal cramp, abdominal tenderness, abdominal pressure, abdominal discomfort, abdomen pain, abdomen cramp, abdomen tenderness, abdomen pressure, abdomen discomfort, left lower quadrant pain, left upper quadrant pain, right lower quadrant pain, right upper quadrant pain, pain abdomen, pain abdominal, pain lower right side of the tummy, pain lower left side of the tummy, pain upper right side of the tummy, pain upper left side of the tummy |
| Contraction | preterm contraction, uterine contraction, contraction |

* Also search variations (adjective, adverb, plural form, and verb tenses) of these words, where applicable. The corresponding misspelled or abbreviated words in table A3 were not listed here.

Table A5. Keywords or phrases and priority used for cervical length extraction.

| **Priority** | **Keywords or phrases*** |
| --- | --- |
| First | transvaginal ultrasound cervix length, transvaginal cervix length, cervix length, length of cervix, measure of cervix, long of cervix, length of the cervix, measure of the cervix, long of the cervix, short cervix, cervix (0-2) of word token length, cervix (0-2) of word token measure, cervix (0-2) of word token short, cervix (0-2) of word token long |
| Second | transvaginal ultrasound cervix, transvaginal cervix, transvaginal, bedside ultrasound, beside sono, endovaginal ultrasound, endovaginal sono |
| Third | cervix exam, cervical exam, cervix change, cervical change, cervix |

* Also search variations (adjective, adverb, plural form, and verb tenses) of these words, where applicable. The corresponding misspelled or abbreviated words in table A3 were not listed here.

“(0-2) of word token” standards zero to two any word token.
